# Repeated Withdrawal of a GLPR Agonist Induces Hyperleptinemia and Deteriorates Metabolic Health in Obese Aging UM‐HET3 Mice

**DOI:** 10.1111/acel.70210

**Published:** 2025-09-17

**Authors:** Nisi Jiang, Jiyuan Yin, Noah Lawrence, Jieyi Meng, Laurence T. Maeyens, Ziying Xu, Xin Li, Mbolle Ekane, Ariana Chaudhary, Pengju Cao, Guannan Li, Carolina Solis‐Herrera, Yi Zhu, Shangang Zhao

**Affiliations:** ^1^ Sam and Ann Barshop Institute for Longevity and Aging Studies University of Texas Health Science Center at San Antonio San Antonio Texas USA; ^2^ Children's Nutrition Research Center, Department of Pediatrics Baylor College of Medicine Houston Texas USA; ^3^ Division of Endocrinology, Department of Medicine University of Texas Health Science Center at San Antonio San Antonio Texas USA

**Keywords:** leptin, liraglutide, repeated withdrawal, sarcopenia, weight cycling

## Abstract

GLP‐1‐based therapy is highly effective in combating aging‐associated metabolic diseases. However, the metabolic effects of frequent withdrawal from this therapy in aged, obese mice have not been previously studied. In this study, aged obese UM‐HET3 mice were assigned to three groups: Group 1 received no liraglutide treatment (Lira OFF); Group 2 underwent 3 cycles of treatment followed by withdrawal (Lira ON/OFF); and Group 3 remained on continuous treatment (Lira ON). As expected, mice in Group 3 showed reduced body weight and food intake, along with improved metabolic health. In contrast, mice in Group 2 developed hyperleptinemia and visceral fat expansion, leading to impaired metabolic health. Importantly, although these mice regained their fat mass after each treatment cycle, they failed to restore lean mass, an unfavorable shift in body composition that may increase vulnerability to aging‐related sarcopenia. These findings suggest that continuous GLP‐1‐based therapy is necessary to sustain metabolic benefits, while intermittent use may promote age‐associated sarcopenia and metabolic decline.

## Graphical abstract

Repeated withdrawal of GLP‐1R agonist drives hyperleptinemia, resulting in visceral fat expansion and impaired metabolic health, which increases the risk of development of aging‐associated sarcopenia.

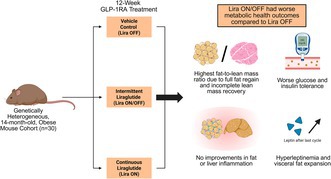

## 1 Introduction

Obesity is closely associated with multiple metabolic disorders, including insulin resistance, type 2 diabetes, metabolic dysfunction‐associated steatotic liver disease (MASLD), cardiovascular disease, and some types of cancers (Drucker 2021; Klein et al. 2022; Reinisch et al. 2025; Zhu et al. 2022). Clinical observations have indicated that even modest weight loss, as low as 5%, can significantly improve metabolic health, reducing the risk of type 2 diabetes and heart disease and improving blood pressure (Varady et al. 2022). As a result, identifying effective strategies to promote substantial weight loss remains a major focus of scientific research.

Compared to lifestyle interventions, which typically achieve up to 10% weight loss (Ard et al. 2024), gastric bypass surgery results in 30%–40% weight loss and is considered the gold standard for obesity treatment. More recently, the successful development of GLP‐1‐based therapies, such as semaglutide (Weghuber et al. 2022) and tirzepatide (Jastreboff et al. 2025), has led to weight loss outcomes in clinical settings that approach those of bariatric surgery (Drucker 2025a, 2025b; Kusminski et al. 2024; Muller et al. 2022). These therapies not only induce significant weight loss but also markedly improve glucose tolerance and insulin sensitivity, while reducing MASLD, liver fibrosis, and the risk of cardiovascular events in both rodents and humans. Given their effectiveness and safety profile, GLP‐1‐based therapies are rapidly becoming the preferred option for weight loss intervention in obese individuals (Finan et al. 2025; Ryan et al. 2024).

However, GLP‐1‐based therapies come with side effects such as nausea, vomiting, diarrhea, and abdominal pain (Borner et al. 2022). In addition, the high cost of treatment poses a significant barrier. These factors lead some individuals with obesity to repeatedly discontinue and resume GLP‐1 therapy, resulting in rapid weight loss followed by rapid weight gain, a distinct pattern of weight cycling. Despite their widespread clinical use, the metabolic consequences of repeated withdrawal from GLP‐1‐based therapies remain poorly understood in both rodents and humans.

To address this gap, we utilized the genetically diverse UM‐HET3 mouse model, which better mimics human genetic heterogeneity and enhances translational relevance. Widely used in aging research, UM‐HET3 mice have been instrumental in identifying interventions that extend lifespan and improve metabolic health through the National Institute of Aging's Intervention Testing Program (ITP) (Miller et al. 2024). In this study, we leveraged this mouse model to investigate the metabolic effects of GLP‐1‐based therapy and its repeated withdrawal. Eight‐month‐old UM‐HET3 mice were placed on a high‐fat diet for 6 months, after which a subset (Lira ON/OFF group) underwent 3 cycles of liraglutide treatment and withdrawal, during which we monitored their metabolic responses. Our findings indicate that aged, obese UM‐HET3 mice subjected to repeated liraglutide withdrawal exhibited impaired metabolic health, developing hyperleptinemia and visceral fat expansion. Notably, during each withdrawal phase, these mice regained fat mass but failed to restore lean mass, an unfavorable shift in body composition that may increase the risk of aging‐associated sarcopenia.

## 2 Results

### 2.1 Repeated Liraglutide Withdrawal Induces Weight Cycling in Aged Obese Mice

Several GLP‐1‐based therapies are currently available, including semaglutide, tirzepatide, liraglutide (Lira), and DPP‐4 inhibitors. In our previous studies, we demonstrated that Lira induces substantial weight loss in diet‐induced obese C57BL/6 mice (Zhao et al. 2024). Based on these findings, we selected Lira to investigate the metabolic effects of repeated withdrawal. Given the shared mechanism of action among GLP‐1 agonists, we expect that semaglutide and tirzepatide would similarly induce weight cycling in mice.

**FIGURE 1:**
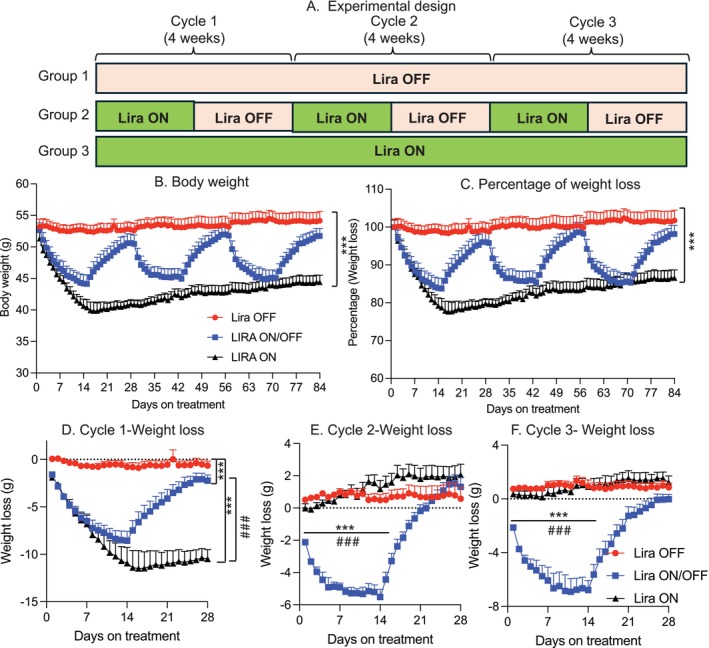
Repeated liraglutide withdrawal induces weight cycling in aged obese mice. (A) Experimental design. (B) Body weight measurements over time in the three groups (*n* = 8–10 mice/group). (C) Percentage of body weight loss (*n* = 8–10 mice/group). (D–F) Absolute weight loss (in grams) during cycles 1, 2, and 3, respectively (*n* = 8–10 mice/group). Data are presented as mean ± SEM. Statistical analyses for repeated measures were analyzed using two‐way ANOVA with Tukey's post hoc test. The statistical significance of (D) was analyzed by separation of Day1 to 144 and Day15 to 288. *p ≤ 0.05, **p ≤ 0.005, ***p ≤ 0.001, ****p ≤ 0.0001 indicates significance compared to the group of Lira OFF; ^#^ *p* ≤ 0.05, ^##^ *p* ≤ 0.005, and ^###^ *p* ≤ 0.001 indicates significance compared to the group of Lira ON.

To better mimic the use of GLP‐1‐based therapy in aging individuals with obesity, we placed 8‐month‐old UM‐HET3 mice on a high‐fat diet for 6 months, creating a genetically heterogeneous, obese, and aging mouse model. The mice were then divided into three groups: Group 1 (Lira OFF) received only vehicle (saline) for three 4‐week cycles; Group 2 (Lira ON/OFF) underwent 3 cycles of 2 weeks of Lira treatment followed by 2 weeks of vehicle; and Group 3 (Lira ON) received continuous Lira treatment for 12 weeks (Figure 1A). Throughout the study, we monitored body weight and food intake during daily Lira injections.

Mice in the Lira OFF group maintained a stable body weight throughout all 3 cycles, showing no weight loss (Figure 1B–C). In contrast, mice in the Lira ON group experienced rapid weight loss during the first cycle and maintained this reduced weight during cycles 2 and 3. Interestingly, mice in the Lira ON/OFF group exhibited rapid weight loss during each treatment phase, followed by rapid weight gain during the withdrawal phases, resulting in three distinct cycles of weight cycling (Figure 1B–C). Despite similar baseline body weights across groups, the Lira ON/OFF group showed a less pronounced weight loss in cycle 1 compared to the Lira ON group, likely due to the genetic heterogeneity of the UM‐HET3 mice. Notably, in the first cycle, Lira treatment led to substantial weight loss that was not fully regained during the withdrawal phase. However, in the second cycle, the weight loss response was attenuated (8 g in cycle 1 vs. 4 g in cycle 2), while the subsequent weight regain was more pronounced. In cycle 3, both weight loss and regain resembled the pattern observed in cycle 2. These results suggest that the efficacy of GLP‐1 agonist treatment diminishes with repeated administration, indicating the potential development of drug resistance.

### 2.2 Repeated Liraglutide Withdrawal Does Not Affect Overall Food Intake

**FIGURE 2:**
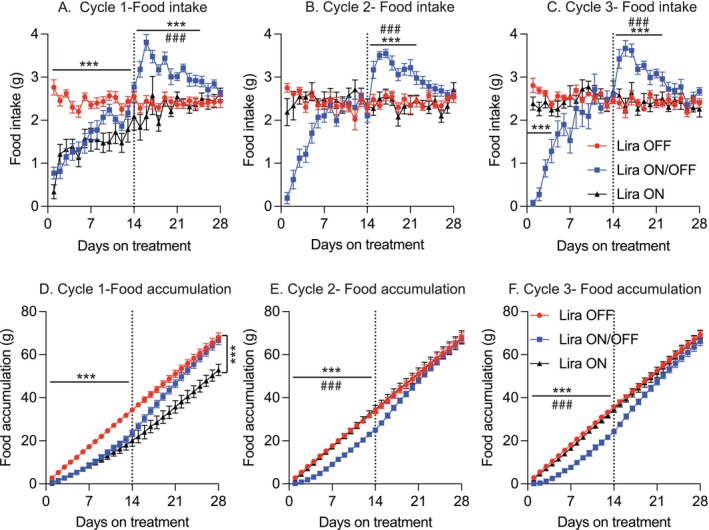
Effects of repeated liraglutide withdrawal on food intake. (A–C) Daily food intake during cycles 1, 2, and 3, respectively (*n* = 8–10 mice/group). (D–F) Total food accumulation over each cycle (*n* = 8–10 mice/group). Data are presented as mean ± SEM. Statistical analyses for repeated measures were analyzed using two‐way ANOVA with Tukey's post hoc test. The statistical significance was analyzed by separation of Day1 to 144 and Day15 to 288. *p ≤ 0.05, **p ≤ 0.005, ***p ≤ 0.001, ****p ≤ 0.0001 indicates significance compared to the group of Lira OFF; ^#^ *p* ≤ 0.05, ^##^ *p* ≤ 0.005, and ^###^ *p* ≤ 0.001 indicates significance compared to the group of Lira ON.

Mice in the Lira OFF group maintained consistent daily food intake from cycle 1 to cycle 3 (Figure 2A–C). In contrast, mice in the Lira ON group exhibited a sharp decline in food intake during the first week of cycle 1, which returned to baseline levels in the second week. Their food intake during cycles 2 and 3 was comparable to that of the Lira OFF group (Figure 2A–C), suggesting that reduced food intake contributed minimally to their sustained lower body weight during these later cycles.

Mice in the Lira ON/OFF group displayed marked fluctuations in food intake: during each Lira treatment phase, they reduced their intake for approximately 1 week before returning to baseline levels. Upon withdrawal of Lira, food intake increased steadily for about 1 week before gradually normalizing (Figure 2A–C). Surprisingly, when we calculated total food accumulation for each cycle, the Lira ON/OFF group consumed the same amount of food as the Lira OFF group (Figure 2D–F).

These findings suggest that the central nervous system maintains tight regulation of long‐term food intake, potentially limiting the effectiveness of appetite‐suppressing interventions over extended periods. This may also help explain the challenges of sustaining weight loss across repeated treatment cycles.

### 2.3 Repeated Liraglutide Withdrawal Differentially Affects Lean Mass and Fat Mass

**FIGURE 3:**
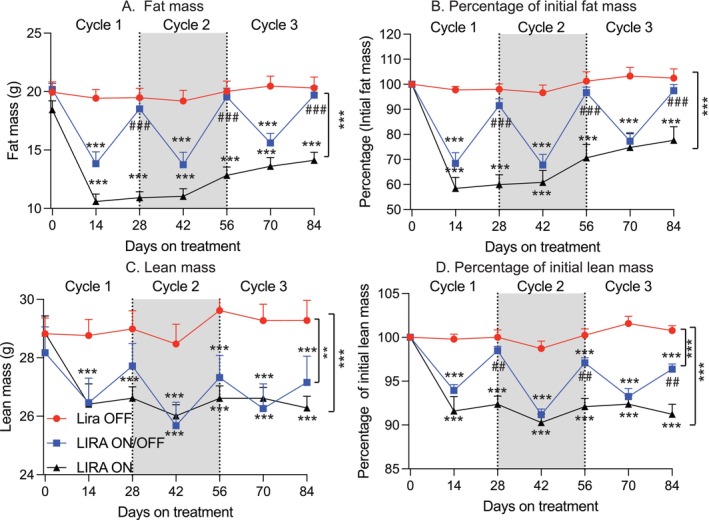
Effects of repeated liraglutide withdrawal on fat mass and lean mass. (A) Total fat mass (g) across cycles 1–3 (*n* = 8–10 mice/group). (B) Fat mass as a percentage of initial fat mass (*n* = 8–10 mice/group). (C) Total lean mass (g) across cycles 1–3 (*n* = 8–10 mice/group). (D) Lean mass as a percentage of initial lean mass (*n* = 8–10 mice/group). Data are presented as mean ± SEM. Statistical analyses for repeated measures were analyzed using two‐way ANOVA with Tukey's post hoc test. In addition, we performed further statistical analysis at each time point from Day 0 to Day 84 using one‐way ANOVA followed by Tukey's post hoc test to indicate the difference at each time point. *p ≤ 0.05, **p ≤ 0.005, ***p ≤ 0.001, ****p ≤ 0.0001 indicates significance compared to the group of Lira OFF; ^#^ *p* ≤ 0.05, ^##^ *p* ≤ 0.005, and ^###^ *p* ≤ 0.001 indicates significance compared to the group of Lira ON.

A major concern with GLP‐1‐based therapy is its potential to reduce lean mass, which may contribute to sarcopenia in aged individuals. In this study, we monitored fat and lean mass every 2 weeks. Mice in the Lira OFF group maintained stable fat mass, both in absolute terms (grams) and as a percentage of initial fat mass (Figure 3A,B). In contrast, mice in the Lira ON group exhibited a significant reduction in total fat mass. However, by cycle 3, a slight rebound in fat mass was observed, suggesting a potential fat rebound effect with prolonged treatment (Figure 3A,B). The most striking findings emerged in the Lira ON/OFF group. Each cycle of Lira treatment resulted in substantial fat mass loss, followed by full restoration of fat mass during the subsequent withdrawal phase (Figure 3A,B). Notably, the fat‐reducing effect of Lira diminished over time, as evidenced by a smaller fat loss in cycle 3 compared to earlier cycles. These results suggest that repeated Lira withdrawal may lead to reduced drug efficacy, potentially reflecting the development of drug resistance in aged, obese mice. As expected, Lira treatment also caused a significant loss of lean mass, measured both in absolute terms and as a percentage of initial lean mass. However, unlike fat mass, lean mass was only partially regained during the withdrawal phases (Figure 3C–D).

Together, these findings indicate that repeated Lira withdrawal fully restores fat mass but fails to restore lean mass, leading to an unfavorable shift in body composition. This imbalance may contribute to the progression of aging‐associated sarcopenia in obese aged mice.

### 2.4 Repeated Liraglutide Withdrawal Impairs Metabolic Health

**FIGURE 4:**
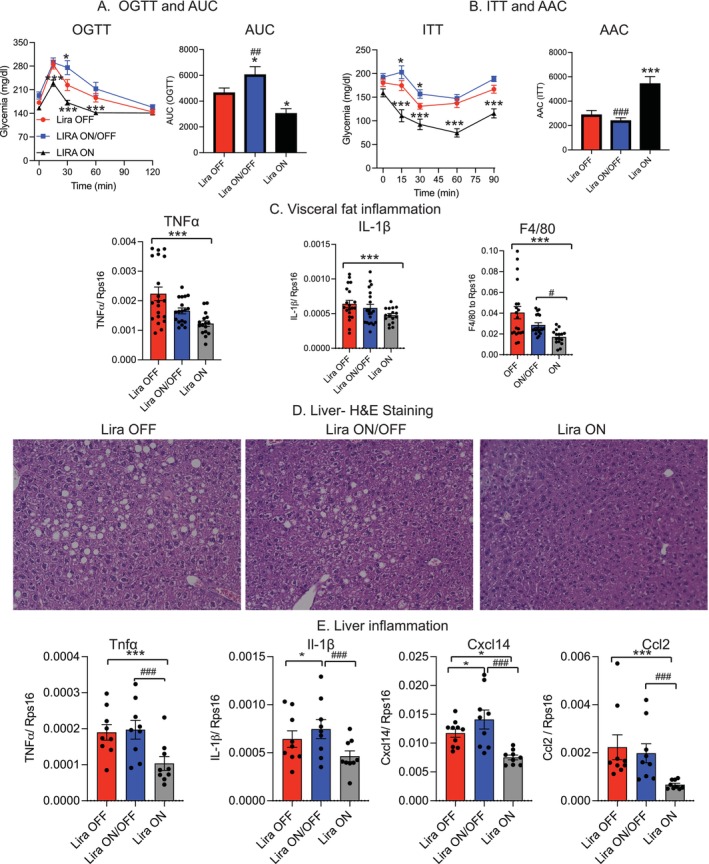
Effects of repeated liraglutide withdrawal on metabolic health. (A) Oral glucose tolerance test (OGTT). (B) Insulin tolerance test (ITT). (C) Expression of inflammatory markers in epididymal visceral fat. (D) Hematoxylin and eosin (H&E) staining of liver sections. (E) Expression of inflammatory markers in liver tissue. Data are presented as mean ± SEM. Statistical analyses for bar graphs were performed using one‐way ANOVA followed by Tukey's post hoc test; *p ≤ 0.05, **p ≤ 0.005, ***p ≤ 0.001, ****p ≤ 0.0001 indicate significance compared to the group of Lira OFF; ^#^ *p* ≤ 0.05, ^##^ *p* ≤ 0.005, and ^###^ *p* ≤ 0.001 indicate significance compared to the group of Lira ON.

Given the observed increase in the fat‐to‐lean mass ratio, we investigated whether repeated Lira withdrawal would further impair metabolic health in these mice. To assess this, we performed glucose and insulin tolerance tests on all three groups at the end of the 12‐week experiment. As expected, mice in the Lira ON group exhibited significantly improved glucose tolerance and insulin sensitivity compared to the Lira OFF group (Figure 4A,B). In contrast, mice in the Lira ON/OFF group, which had a markedly higher fat‐to‐lean mass ratio, showed severely impaired glucose tolerance and insulin sensitivity, as indicated by elevated blood glucose levels following glucose and insulin challenges (Figure 4A,B). Furthermore, we examined markers of inflammation in adipose tissue and liver. Mice in the Lira ON group showed significantly reduced expression of inflammatory markers, including Tnfα, IL‐1β, and F4/80 in visceral adipose tissue (Figure 4C). However, this anti‐inflammatory effect was completely absent in the Lira ON/OFF group, indicating that repeated Lira withdrawal failed to confer any lasting anti‐inflammatory benefit. In addition, histological analysis using H&E staining revealed a substantial reduction in hepatic lipid droplet accumulation in the Lira ON group, an effect that was entirely lost in the Lira ON/OFF group (Figure 4D). Similarly, inflammatory markers in the liver, including Tnfα, IL‐1β, Cxcl14, and Ccl2, were significantly reduced in the Lira ON group but remained elevated in the Lira ON/OFF group (Figure 4E). These observations strongly suggest that repeated Lira withdrawal fails to produce any lasting “metabolic memory” effects.

### 2.5 Cellular Senescence in the Adipose Tissue and Liver Does Not Contribute to the Detrimental Effects of Repeated Liraglutide Withdrawal

Cellular senescence plays a critical role in regulating metabolic health in the context of obesity and aging (Yousefzadeh et al. 2021; Zhang et al. 2022, 2024, 2023). Previous studies have shown a significant increase in cellular senescence within visceral fat depots and the liver during obesity development (Ogrodnik et al. 2017; Palmer et al. 2019). Given the profound effects of Lira on visceral fat and liver tissue, we investigated whether changes in cellular senescence might underlie the observed metabolic outcomes.

**FIGURE 5:**
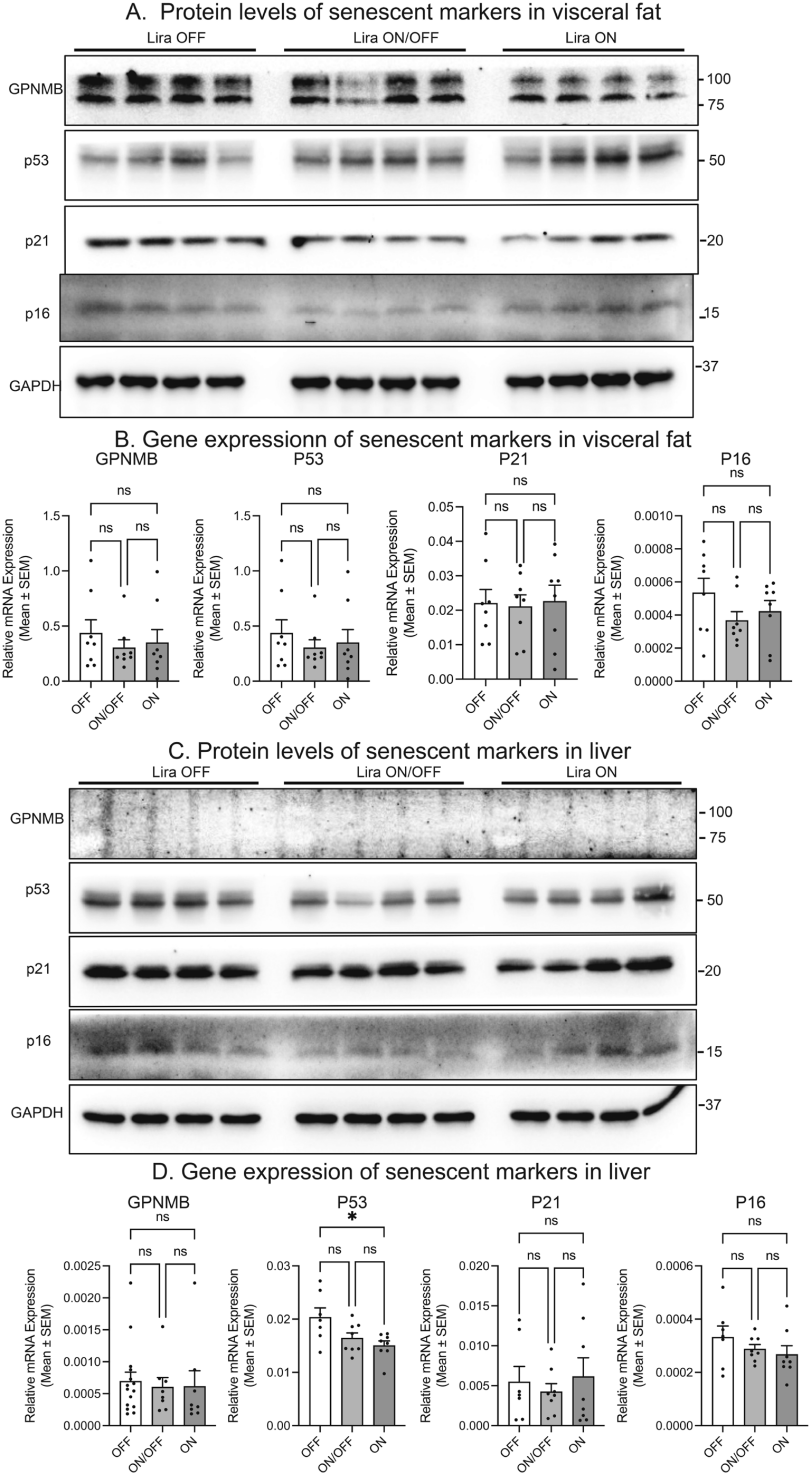
Cellular senescence is not involved in the metabolic effects of liraglutide treatment. (A) Protein levels of key senescence markers in visceral fat depots. (B) Gene expression of senescence markers in visceral fat. (C) Protein levels of senescence markers in liver tissue. (D) Gene expression of senescence markers in liver. Data are presented as mean ± SEM. Statistical analyses for bar graphs were performed using one‐way ANOVA followed by Tukey's post hoc test; *p ≤ 0.05, **p ≤ 0.005, ***p ≤ 0.001, ****p ≤ 0.0001 indicates significance compared to the group of Lira OFF; ^#^ *p* ≤ 0.05, ^##^ *p* ≤ 0.005, and ^###^ *p* ≤ 0.001 indicate significance compared to the group of Lira ON.

To explore this, we analyzed the expression of key senescence markers (GPNMB, p53, p21, and p16) using western blotting and RT‐PCR in both visceral fat depots and liver tissue. Surprisingly, despite the significant reduction in fat mass observed in the Lira ON group, we did not detect any changes in the expression of senescence markers in visceral fat (Figure 5A,B). Similarly, in the Lira ON/OFF group, which underwent repeated cycles of fat loss and regain, senescence marker expression remained unchanged (Figure 5A,B). In the liver, improved steatosis in the Lira ON group was not accompanied by reduced senescence markers. Similarly, the Lira ON/OFF group showed no differences compared to the Lira OFF and ON groups (Figure 5C,D). These findings suggest that adipose and liver senescence do not mediate the metabolic effects of repeated Lira withdrawal, though other tissues like muscle, pancreas, and gut warrant further investigation.

### 2.6 Repeated Liraglutide Withdrawal Promotes Hyperleptinemia Driving Metabolic Impairment

We previously demonstrated that hyperleptinemia contributes to diet‐induced obesity and that leptin reduction is essential for GLP‐1‐induced weight loss (Zhao, Kusminski, et al. 2020; Zhao et al. 2021, 2023, 2019; Zhao, Li, et al. 2020). Here, we investigated whether leptin plays a role in the metabolic impairments observed with repeated Lira withdrawal. To this end, we measured circulating insulin, adiponectin, and leptin before, during, and after the treatment.

**FIGURE 6:**
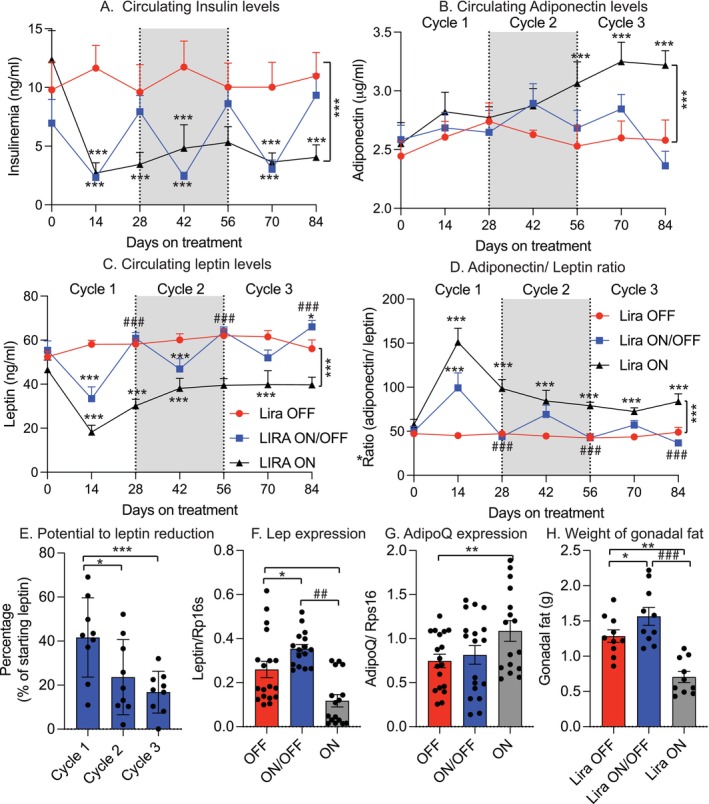
Effects of repeated liraglutide withdrawal on circulating insulin, adiponectin, and leptin. (A) Circulating insulin levels across cycles 1–3. (B) Circulating adiponectin levels across cycles 1–3. (C) Circulating leptin levels across cycles 1–3. (D) Adiponectin/leptin ratio over time. (E) Magnitude of leptin reduction from cycle 1 to cycle 3. (F) *Lep* gene expression in epididymal fat. (G) *AdipoQ* gene expression in epididymal fat. (H) Epididymal visceral fat depot weight. Data are presented as mean ± SEM. Statistical analyses for repeated measures were analyzed using two‐way ANOVA with Tukey's post hoc test. In addition, for (A), we performed further statistical analysis at each time point from Day 0 to Day 84 using one‐way ANOVA followed by Tukey's post hoc test to indicate the difference at each time point. Statistical analyses for bar graphs were performed using one‐way ANOVA followed by Tukey's post hoc test; *p ≤ 0.05, **p ≤ 0.005, ***p ≤ 0.001, ****p ≤ 0.0001 indicates significance compared to the group of Lira OFF; ^#^ *p* ≤ 0.05, ^##^ *p* ≤ 0.005, and ^###^ *p* ≤ 0.001 indicates significance compared to the group of Lira ON.

We measured circulating insulin, adiponectin, and leptin before, during, and after treatment. Lira treatment significantly reduced insulin levels (Figure 6A). In the Lira ON/OFF group, insulin levels fluctuated with treatment: they decreased during Lira administration and returned to baseline upon withdrawal, matching the Lira OFF group. Adiponectin levels showed no consistent pattern and appeared to require prolonged treatment for elevation, only increasing after 56 days in the Lira ON group (Figure 6B). The Lira ON/OFF group showed no significant change compared to Lira OFF, suggesting adiponectin is stable and unresponsive to short‐term weight fluctuations. Leptin, however, displayed distinct dynamics. Mice in the Lira OFF group maintained steady leptin levels throughout the study (Figure 6C). In contrast, the Lira ON group showed a marked decrease in leptin during cycle 1, followed by a gradual increase in cycles 2 and 3, despite only modest weight regain. Most notably, the Lira ON/OFF group exhibited a progressive rise in circulating leptin, reaching hyperleptinemia after three treatment cycles. We believe this rise likely contributed to the observed metabolic impairments. We also analyzed the adiponectin/leptin ratio, a recognized marker of metabolic health, and found that after an initial increase it declined over time in both Lira ON and Lira ON/OFF groups. By the third cycle, the ratio in the Lira ON/OFF group was even lower than in the Lira OFF group, consistent with the impaired metabolic outcomes observed in this group (Figure 6D).

Since leptin reduction is necessary for GLP‐1‐induced weight loss, we calculated the percentage decrease in leptin across cycles. The extent of leptin reduction diminished from cycle 1 to cycle 3 (Figure 6E), mirroring the reduced weight loss over time.

To investigate the source of hyperleptinemia, we assessed Lep gene expression in visceral fat. *Lep* expression was highest in the Lira ON/OFF group (Figure 6F), while *AdipoQ* expression remained unchanged (Figure 6G). Additionally, visceral fat mass was significantly greater in the Lira ON/OFF group compared to Lira OFF by the end of the third cycle (Figure 6H), supporting the link between hyperleptinemia and visceral fat expansion.

## 3 Discussion

Using aging, obese, and genetically heterogeneous UM‐HET3 mice, a model that better reflects the complexity of human populations, we made several key observations regarding the effects of repeated liraglutide withdrawal. Compared to continuous administration, repeated cycles of liraglutide treatment and withdrawal induced what appears to be a form of drug tolerance, evidenced by a progressive reduction in weight loss and fat mass reduction over time. This diminished efficacy was accompanied by amplified gastrointestinal side effects, as shown by a progressively lower food intake in response to acute liraglutide administration from cycle 1 to cycle 3. Importantly, repeated withdrawal impaired overall metabolic health and appeared to increase the risk of developing obesity‐ and aging‐associated sarcopenia. These detrimental outcomes were likely mediated by progressively increasing leptin levels or hyperleptinemia. Given that all GLP‐1 receptor agonists, including semaglutide, tirzepatide, and liraglutide, act primarily through Glp1r‐expressing neurons in the brain to regulate satiety and food intake, we expect that the effects observed with liraglutide in this study also apply broadly to other GLP‐1R agonists.

Although weight cycling has been studied extensively in contexts such as dietary restriction and refeeding (Bernecker et al. 2025; Zamboni et al. 2025), GLP‐1‐induced weight cycling appears mechanistically distinct. One major concern associated with GLP‐1‐based therapies is the loss of lean mass, which has been well documented in clinical studies. In our model, liraglutide administration led to marked reductions in both fat and lean mass. However, while fat mass was fully restored during withdrawal, lean mass recovery was only partial. This disproportionate regain of fat relative to lean tissue suggests that individuals who discontinue GLP‐1 therapy may be at increased risk of developing sarcopenia, compared to those who never initiate treatment. These findings raise important clinical considerations, as they suggest that intermittent use of GLP‐1‐based therapies could exacerbate metabolic vulnerability rather than improve it. Thus, long‐term commitment to treatment may be necessary to sustain benefits, and clinicians should carefully weigh these long‐term implications when prescribing these therapies.

To explore the mechanisms underlying the detrimental effects of repeated withdrawal, we considered whether repeated weight loss and regain in adipose tissue might induce cellular senescence, leading to inflammation and other metabolic dysfunctions. However, our analyses of senescence markers in both adipose tissue and liver revealed no significant differences across treatment groups, suggesting that cellular senescence in these tissues is not the primary driver of the observed metabolic impairments. Still, this does not exclude the possibility that senescence in other tissues, such as skeletal muscle, pancreas, or gut, contributes to impaired lean mass recovery and the development of sarcopenia. In contrast, our findings point strongly toward altered adipokine signaling, particularly involving leptin, as a key mediator of the observed effects. We and others have previously shown that hyperleptinemia is not only a biomarker but also a causal factor in diet‐induced obesity (Pretz et al. 2021; Zhao et al. 2019). Moreover, leptin reduction is necessary for the weight‐lowering effects of GLP‐1 therapy (Zhao et al. 2024). Our current results are consistent with this model: repeated liraglutide withdrawal led to progressively diminished reductions in circulating leptin, paralleling the diminished weight loss over successive cycles. Additionally, we observed increased *Lep* gene expression in visceral fat and elevated circulating leptin levels, which likely drove visceral fat expansion through paracrine or autocrine mechanisms. This expansion of visceral fat occurred despite stable or only slightly increased overall body weight, suggesting a shift in body composition that favors adiposity over lean tissue. Recent studies suggest that adipocytes may retain an “obesogenic memory,” priming them for pathological responses upon re‐exposure to obesogenic stimuli (Hinte et al. 2024). Our observations during liraglutide withdrawal, characterized by rapid fat regain, sustained hyperleptinemia, and impaired metabolic markers, are consistent with this concept and suggest that altered adipokine secretion, including persistently elevated leptin, may underlie such memory effects.

A high dropout rate has been documented in real‐world populations using GLP‐1R agonists (Endo and Kami 2025). However, the metabolic consequences of frequent treatment discontinuation have not been systematically studied in human populations. Future research is needed to evaluate whether repeated withdrawal in humans leads to similar risks, including sarcopenia and altered adipokine profiles.

In summary, our findings demonstrate that repeated liraglutide withdrawal promotes hyperleptinemia, impairs metabolic health, and accelerates the development of aging and obesity associated sarcopenia. These results suggest that continuous, rather than intermittent, use of GLP‐1‐based therapy should be considered from the outset to ensure sustained metabolic benefits.

## 4 Materials and Methods

### 4.1 Mouse Models

All animal procedures were approved by the Institutional Animal Care and Use Committee (IACUC) of the University of Texas Health Science Center at San Antonio. Male UM‐HET3 mice (4–5 per cage) were obtained through the NIA‐sponsored Intervention Testing Program (ITP) at the San Antonio site. Breeding methods have been described previously and are summarized here briefly (Miller et al. 2024). UM‐HET3 mice were the progeny of CByB6F1 mothers (JAX stock #100009) and C3D2F1 fathers (JAX stock #100004). Mice from second or later litters, produced over a six‐month interval, were weaned into cages of 4–5 males and maintained under standard laboratory conditions (12‐h light/dark cycle; lights on at 7:00 a.m.) in a temperature‐controlled (22°C) environment with ad libitum access to food and water.

### 4.2 Mouse Treatment

At 8 months of age, mice were placed on a high‐fat diet (Bio‐Serv, 60% fat) for 6 months. Mice with comparable body weight and food intake were then assigned to three groups and administered daily intraperitoneal injections of either vehicle (saline) or liraglutide (0.01 mg/kg body weight). Body weight and food intake were monitored throughout the treatment period. Body composition was assessed using EchoMRI. The same experiments have been repeated two times.

### 4.3 Oral Glucose and Insulin Tolerance Tests

Following the three treatment cycles, oral glucose tolerance tests (OGTT) and intraperitoneal insulin tolerance tests (ITT) were performed as previously described at the end of 12 weeks' treatment (Zhao et al. 2014). Mice were then euthanized, and blood was collected from the inferior vena cava. Livers were perfused with PBS, and liver and adipose tissues were dissected and weighed. Blood samples were allowed to clot at room temperature, centrifuged at 2000 × *g* for 10 min at 4°C, and the serum supernatant was flash‐frozen in liquid nitrogen and stored at −80°C. Tissue samples were either snap‐frozen or fixed in formalin for further analysis. Serum insulin, adiponectin, and leptin were measured using ELISA kits (Crystal Chem).

### 4.4 RNA Isolation, RT‐qPCR and RNAseq

RNA extraction and analysis were performed as previously described (Zhao et al. 2025). In brief, total RNA was extracted using TRIzol reagent (Invitrogen, Cat# 15596026) and purified by isopropanol precipitation. RNA concentration was determined using a NanoDrop spectrophotometer (Thermo Scientific). For RT‐qPCR, 1 μg of RNA was reverse‐transcribed using the Hiscript III 1st Strand cDNA Synthesis Kit with gDNA wiper (Vazyme, R312). Gene expression was quantified on a CFX384 Real‐Time PCR Detection System (Bio‐Rad) using the ΔΔCt method and normalized to 16S rRNA expression.

### 4.5 Histology

Liver tissue was harvested, fixed in 10% phosphate‐buffered formalin (Fisher Chemical, Cat# SF100‐20) for 2 weeks at room temperature, and embedded in paraffin. Sections (4 μm) were cut and mounted on glass slides. Hematoxylin and Eosin (H&E) staining was performed on liver tissue sections as described previously (Li et al. 2023), and images were acquired using a BZ‐X800E microscope (KEYENCE).

### 4.6 Statistical analysis

Results are shown as mean ± SEM. Statistical analyses for repeated measures with three groups were analyzed using two‐way ANOVA with Turkey's post hoc test. In addition, to compare the difference with two groups, statistical analysis was performed using one‐way ANOVA followed with Tukey's post hoc test to indicate the difference. * was used to indicate the difference to LIRA OFF group; # was used to indicate the difference to the LIRA ON group. Also, *p*‐values < 0.05 were considered statistically significant. *p < 0.05; **p < 0.01; ***p < 0.001; #*p* < 0.05; ##*p* < 0.01; ###*p* < 0.001. Additional details were provided in figure legends.

## Author Contributions

N.J. and S.Z. designed the study. N.J., N.L., J.Y., and S.Z. analyzed the results. G.L. and S.Z. drafted the manuscript. G.L., J.Y., N.L., J.M., L.T.M, Z.X., X.L., M.E., A.C., P.C. contributed to the acquisition and analysis of data. G.L., C.S.‐H., Y.Z., L.T.M, M.E., and S.Z. revised and approved the final version of the manuscript. S.Z. is the guarantor of this work, with full access to all data and analyses related to the content of this article.

## Conflicts of Interest

The authors declare no conflicts of interest.

## Data Availability

The data that support the findings of this study are available from the corresponding author upon request.
